# Toward a Mixed-Methods Research Approach to Content Analysis in The Digital Age: The Combined Content-Analysis Model and its Applications to Health Care Twitter Feeds

**DOI:** 10.2196/jmir.5391

**Published:** 2016-03-08

**Authors:** Eradah O Hamad, Marie Y Savundranayagam, Jeffrey D Holmes, Elizabeth Anne Kinsella, Andrew M Johnson

**Affiliations:** ^1^ Department of Psychology Faculty of Arts and Humanities King Abdulaziz University Jeddah Saudi Arabia; ^2^ Health and Rehabilitation Sciences Graduate Program Faculty of Health Sciences Western University London, ON Canada; ^3^ School of Health Studies Western University London, ON Canada; ^4^ School of Occupational Therapy Western University London, ON Canada

**Keywords:** health care social media, Twitter feeds, health care tweets, mixed methods research, content analysis, coding, computer-aided content analysis, infodemiology, infoveillance, digital disease detection

## Abstract

**Background:**

Twitter’s 140-character microblog posts are increasingly used to access information and facilitate discussions among health care professionals and between patients with chronic conditions and their caregivers. Recently, efforts have emerged to investigate the content of health care-related posts on Twitter. This marks a new area for researchers to investigate and apply content analysis (CA). In current infodemiology, infoveillance and digital disease detection research initiatives, quantitative and qualitative Twitter data are often combined, and there are no clear guidelines for researchers to follow when collecting and evaluating Twitter-driven content.

**Objective:**

The aim of this study was to identify studies on health care and social media that used Twitter feeds as a primary data source and CA as an analysis technique. We evaluated the resulting 18 studies based on a narrative review of previous methodological studies and textbooks to determine the criteria and main features of quantitative and qualitative CA. We then used the key features of CA and mixed-methods research designs to propose the combined content-analysis (CCA) model as a solid research framework for designing, conducting, and evaluating investigations of Twitter-driven content.

**Methods:**

We conducted a PubMed search to collect studies published between 2010 and 2014 that used CA to analyze health care-related tweets. The PubMed search and reference list checks of selected papers identified 21 papers. We excluded 3 papers and further analyzed 18.

**Results:**

Results suggest that the methods used in these studies were not purely quantitative or qualitative, and the mixed-methods design was not explicitly chosen for data collection and analysis. A solid research framework is needed for researchers who intend to analyze Twitter data through the use of CA.

**Conclusions:**

We propose the CCA model as a useful framework that provides a straightforward approach to guide Twitter-driven studies and that adds rigor to health care social media investigations. We provide suggestions for the use of the CCA model in elder care-related contexts.

## Introduction

### Overview

In the digital age, social networking sites such as Twitter are increasingly turned to as an information source, as they offer a large amount of digital text and are readily available to multisite apps (eg, personal computers, mobile phones, and tablets). Health discussions, for example, occur regularly on Twitter, with online discussions and content sharing among a variety of populations, including health care professionals, patients with chronic conditions, and their caregivers. Some efforts have emerged to investigate the content of health care-related posts on Twitter, constituting a new area for researchers to investigate using content analysis (CA). These approaches are also known as infodemiology, infoveillance, or digital disease detection research. In many of these research initiatives, quantitative and qualitative Twitter data are combined, but there are few clear guidelines for researchers or reviewers to follow when collecting and evaluating this content. An explanation for this could be that contemporary CA is best described as a juxtaposition of quantitative (eg, frequency analysis to count words in a text and represent them statistically) and qualitative (eg, nonfrequency analysis for in-depth hermeneutic interpretations of a text) methodological dimensions [1]. Whether CA should be approached quantitatively or qualitatively has been debated in the literature since modern CA originated in the 1930s [2]. However, these approaches (quantitative and qualitative [3,4]) to CA have common features, including the sampling and data collection strategy (defining the source and amount of content to be collected for analysis), the coding process (defining the units of analysis, training coders, and establishing the coding scheme), and validation of study results (assessing reliability and validity or trustworthiness of study results). These key features of CA may vary according to research aims [1,4-8].

Research using social media platforms (eg, Facebook, Twitter, or LinkedIn) is in the early stages, and despite the great potential for the application of CA to Twitter-based health care content, there are few guidelines for the collection, analysis, and evaluation of the various types of Twitter data. Thus, the aim of our study was to use criteria available in the CA literature, specifically literature on the use of CA in health care research, to identify and evaluate published studies that used Twitter as a primary source of data and CA as a method of analysis and interpretation. Based on our analysis, we propose the combined content-analysis (CCA) model as an organizing framework to guide the application of integrated methods (quantitative and qualitative) and modes (manual and computer assisted) of CA, and to address the varied nature of Twitter feed data (eg, textual, numerical, audio, and video material) within single or multiple-phase studies.

In this paper, we first discuss the position of CA in previous research and then illustrate how CA has been used in health care research. Building on common characteristics of CA found in the literature, we evaluate 18 studies published between 2010 and 2014. Finally, we propose the CCA model of CA along with mixed-methods research approaches. We suggest how to apply the CCA model and offer supporting resources drawing on elder care-related examples.

### Background

#### Positioning of CA

CA is a research methodology or set of methods to analyze content collected from written (eg, open-ended surveys, personal communications, letters, diaries, short stories, newspapers or magazines, and theoretical or methodological trends in journal papers), verbal (eg, interviews, focus groups, radio programs, and folk songs), or visual (eg, films, videos, and TV programs) materials, from printed and electronic resources [2,7,9]. In the digital age, CA may also be used to analyze digital texts (eg, Web-published news, Internet forums, and social media discussions). Once the research aim is stated and the source of data (content components) is identified, data may be sampled and subjected to either qualitative or quantitative analysis, or both. The process of CA consists of coding raw data (eg, papers, interview transcripts, or images) according to a developed or predefined classification scheme (a coding manual). Both qualitative and quantitative approaches can be applied to analyze targeted material. The appropriate method(s) to collect, analyze, and classify content is a critical choice that needs to take careful account of many methodological considerations based on the intended application of CA to the proposed study.

Between the 1930s and 1950s, CA was called “symbol analysis” and was a scientific method of recording the frequency of certain keywords found in newspapers [2]. Cartwright [10] was the first to propose CA and coding as interchangeable terms. When understood this way, CA is viewed as a quantitative approach, whereby text data are coded into categories (code frequencies) based on pre-existing knowledge or hypotheses and then described using codebooks and statistical techniques that allow for future inferences [3,7]. According to Berelson [11], CA is an objective and systematic description of the manifest content. Quantitative concepts have historically been essential to CA. These concepts include objectivity, systematicity, generalizability, transferability, validity, and reliability. In addition, this view of CA requires well-defined samples and units of analysis and stability of results across coders and over time [3]. The quantitative perspective of CA emphasizes the “objective” and consistent quantification or classification of categorical (“subjective”) data [12]. However, some scholars deemed this approach simplistic, arguing that it was not conducive to detailed statistical analysis [7]. Restricting CA to numerical values and the frequency of symbols and other units may create theoretical and practical problems [9,13,14].

As CA spread to other disciplines in the social sciences, such as sociology, psychology, business, and health research, the qualitative approach to CA was developed and was recognized as an approach for data analysis in many research disciplines [7,15,16]. Researchers using qualitative CA may go beyond counting the frequency of words in a text and focus more on the context, including the analyst’s assumptions, preunderstandings, or constructions of reality, the conceptual environment, and where the text is situated in relation to other studies. Context can be construed in relation to the personal or social constructs that support researchers’ questions [17]; thus, qualitative CA may differ across fields of study and from one content analyst to another. In contrast, some researchers argue that CA is insufficiently qualitative and presents some methodological obstacles [9,13,16]. Still others argue that the reading of a text may not differ between researchers and nonresearchers (eg, public readers or study participants). The importance of the description of context related to qualitative CA can also be applied to Twitter as a public data source of social networking and communication, where richness of data, such as user information and biographies and social networking communication (eg, information about “following” and “number of followers”, Twitter chat managers, and community), is as important as the exploration of the content of tweets.

CA researchers such as Holsti [9], Krippendorff [7], and Schreier [6] are generally in agreement that qualitative and quantitative CA are not discrete classifications, but rather fall along a continuum. Consequently, moving back and forth between these approaches affords a greater opportunity to gain insight into the meaning of data [9]. Similarly, Pool [18] suggested that these seemingly opposite approaches to CA exist within a feedback loop in which each approach provides new insights upon which the other can feed. Accordingly, one should not assume that qualitative methods are insightful or that quantitative methods are merely mechanical methods to check hypotheses. By definition, CA is a research approach that can be situated at the intersection of quantitative and qualitative methods, a place where both methods can meet [2] and that quantifies and qualifies the manifest and latent meanings of the data [19]. However, we argue that researchers need to consider combining this understanding of CA with a solid mixed-methods design, especially with the massive growth of digital texts and multimedia data.

#### CA in Health Care Research

CA has come into widespread use in health care research in recent years because of its sensitivity and flexibility as a research technique concerned with meanings, intentions, consequences, and context [15,20]. A review of health studies literature using the Cumulative Index to Nursing and Allied Health Literature shows that the use of CA increased, being mentioned in 97 papers in 1991 and rising to 601 in 2002 [20]. A similar review of nursing studies by Elo and Kyngäs [15] found that the analysis process remains challenging for health care researchers regardless of the flexibility of CA, because there are no clear guidelines for its use. Elo and Kyngäs [15] highlighted the heterogeneity of CA research, noting that it has been mostly used as a general qualitative method for research on psychiatry (713 papers), health care (627 papers), nursing (625 papers), gerontologic care (441 papers), public health (389 papers), and occupational therapy (165 papers).

Hsieh and Shannon [20] divide qualitative CA into three distinct approaches: conventional CA or the “inductive approach” [15]; directed CA or the “deductive approach” [15]; and summative CA or the “manifest approach” [20]. According to Hsieh and Shannon [20], all three approaches adhere to the naturalistic paradigm and can be used to interpret meaning from the content of text data. In addition, the three approaches require a similar analytical process consisting of 7 steps: (1) formulating the research questions, (2) selecting the sample for analysis, (3) defining the categories to be applied, (4) outlining the coding process and training the coders, (5) implementing the coding process, (6) determining trustworthiness, and (7) analyzing the results of the coding process ([20], p 1285). The key differences between these approaches are the initial codes developed by the coder(s), which are generally determined according to the purpose of the study. The intended approach can guide coding schemes and affect the study’s trustworthiness (the quality criteria of qualitative research).

In conventional CA, it is assumed that because there is insufficient or fragmented knowledge about a phenomenon [15], categories and their content are data driven [1]. In this case, the researcher starts the analysis without predetermined categories (eg, theory or concept driven) and derives categories inductively during data analysis. Using this approach, the researcher gains a rich understanding of the phenomenon under investigation, as new insights emerge from the study results. Elo and Kyngäs [15] suggested that a CA approach based on inductive data can be used if the researcher aims to develop a theory, as this approach allows him or her to move from specific details to the general picture of the phenomenon. For example, in Juvani and colleague’s [21] qualitative study, they developed two categories inductively from participants’ interviews to describe the threats and supportive aspects of the physical environment for the well-being of adults over the age of 65 years.

Deductive or directed CA can be used when the purpose of the study is to test a theory or extend an existing theory or prior research [1,15,20]. In a directed approach, categories are determined prior to data analysis. The researcher’s role is to examine and code the data according to these corresponding categories. Thus far, the qualitative deductive approach has been applied infrequently in health research; as such, studies are typically based on an earlier review of the literature, theory, or model, moving from the general to the specific [15]. For example, Latvala et al [22] applied a deductive CA to examine three predefined categories related to psychiatric patients’ participation in their care in a hospital environment. However, Kondracki et al [1] argued that inductive and deductive approaches to CA are not mutually exclusive and can be mixed in a single study. According to Kondracki et al [1], one way to accomplish this integration is to augment quantitative CA by conducting an initial qualitative analysis. Alternatively, the results of qualitative CA can be used to refine quantitatively derived categories and, if necessary, create new variables to capture new aspects of content.

**Figure 1:**
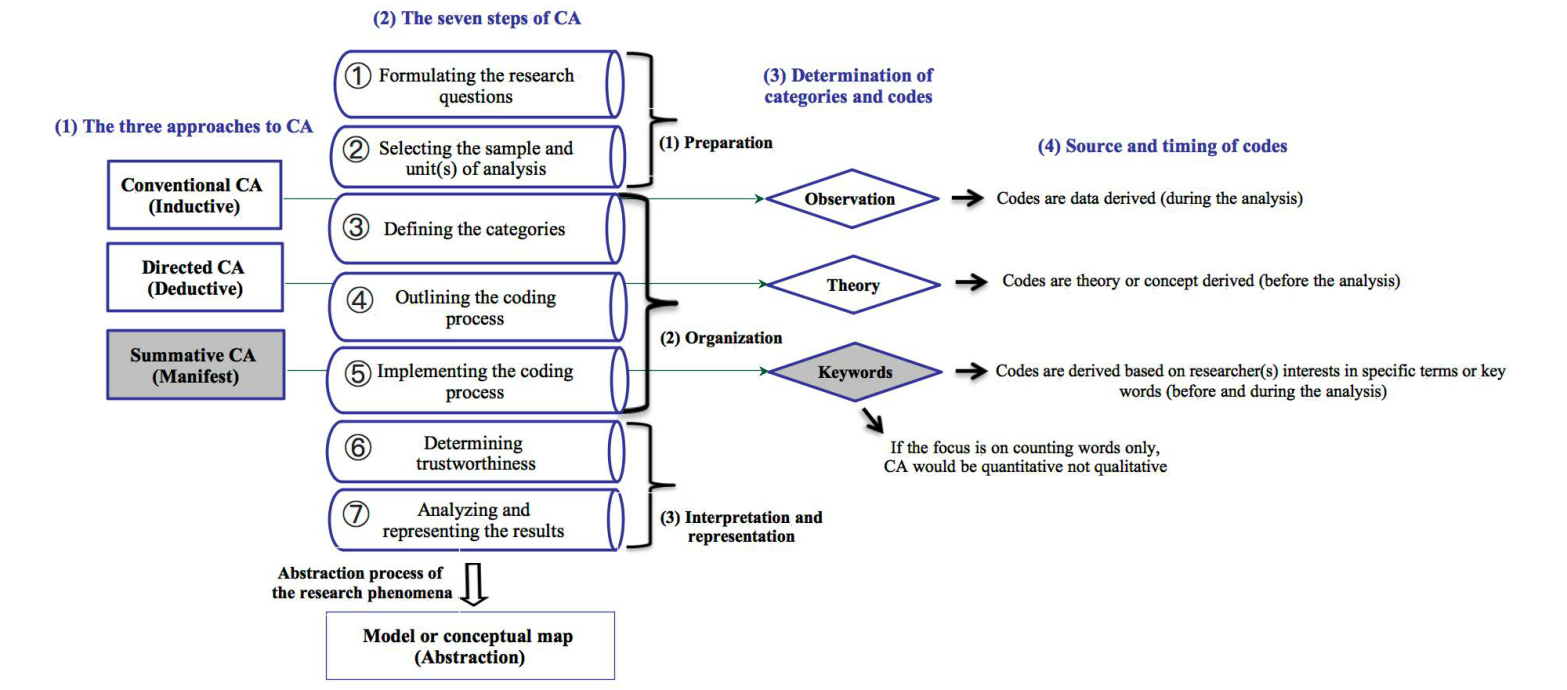
Content analysis (CA) in health care research. Adapted from Hsieh & Shannon (2004, p 1286, Table 4) with permission of SAGE Publications, Inc.

The third type of CA used in health care research is the summative approach. Rather than the data being analyzed as a whole, as in the previous two approaches, the text is searched for particular words or content in relation to a particular topic. For example, the summative approach was used to examine content related to end-of-life care in 14 critical care nursing textbooks [23] and 50 best-selling medical textbooks [24]. Hsieh and Shannon [20] held that if the analysis were to stop at this point it would be quantitative and focused only on the manifest content. A summative approach to qualitative CA goes beyond counting words to include the latent content, the process of interpreting the content, and the discovery of the underlying meaning and alternative terms for the words. Figure 1 summarizes the three approaches to CA in health care research and their steps.

## Methods

To locate current trends in health care social media studies and studies using CA to analyze data from the most popular social media tool, Twitter, we conducted a PubMed search of the years 2010 to 2014. Keyword sets combined “content analysis” AND one of the following: “healthcare social media,” “social networking websites,” “Twitter-driven content,” “Twitter feeds,” OR “healthcare tweets.” The primary research questions were “How is CA used in health care social media studies?” and “Does it follow the common features of CA literature identified in CA research, in general, and health care-related research, in particular?” Paper selection was based on the title and the abstracts. In case of uncertainty, we read the entire text of a paper. In addition, we manually searched the reference lists of all included studies. From the 21 studies found, we selected 18 for examination (see Table 1 [25-42] for the list of studies of health-related tweets published between 2010 and 2014). We based the evaluation of these papers on the narrative review of CA methodological textbooks [6-9,11,17,18,43,44] and CA in published literature [1-5,13-16,20,45,46]. After examining the papers, we constructed the CCA model, which we explained in detail in the Discussion section.

## Results

Our results show that, in the 18 studies examined (in English), Twitter was used as a public and real-time source for textual health data where users tried to disseminate health information from formal sources (eg, academic journals or news websites) and informal sources (eg, personal opinions or actual experiences). In these studies, researchers analyzed Twitter messages using CA as a sole technique or with other research techniques, such as the infoveillance approach (eg, [25,26]), the cross-sectional survey approach (eg, [27-29]), and discourse analysis (eg, [30]). Our review of these studies showed that the quantitative approach was the most common approach to CA (eg, [25-28,31-38]). In addition, it is clear that researchers neither follow a particular procedural model of data analysis and interpretation, nor use straightforward guidelines that would lead other researchers in their evaluation of social media-driven content. In all studies shown in Table 1, the qualitative summative (manifest CA) approach [20] was used as an initial step to track, archive, or retrieve tweets related to the topic of interest (eg, elder care). By identifying and quantifying certain words (eg, elder care, dementia, or Alzheimer) or hashtags (eg, #eldercare, #dementia, or #Alzheimer) using Twitter’s search function or a Twitter archive software program (see Table 2 for a list of software used in the analyzed studies to archive tweets), researchers were able to access hundreds, thousands, or millions of tweets based on the availability of the target topic, time frame (eg, hours, days, or weeks), and frequency of discussions on Twitter at the time of data collection. Through this process, researchers formed a Twitter database for each topic, generating a data pool from which to select their samples. Because the quantitative approach was the leading approach in most studies, the random sampling technique was commonly used, even when CA was used as a qualitative research technique (eg, [39,40]).

**Table 1:** Studies analyzing health-related Twitter posts (2010–2014).

Author(s)	Keywords and hashtags (#)	Sampling and data collection	Data analysis (coding process)	Validation and presentation of results
Chew & Eysenbach (2010) [25]	“swine flu”, “swineflu”, and “H1N1”	Random sample of 5395 tweets for 9 days (each 4 weeks apart) generated from 2 million archived tweets over 8 months. Tweets were posted between May 1 and December 31, 2009 (n=600 tweets/per day were collected for analysis).	Infoveillance approach (statistical classifier) for tracking flu rate (longitudinal text mining and analysis). This approach includes in-depth qualitative manual coding, automated CA^a^using a triaxial coding scheme, and sentiment analysis.	Pilot coding (1200 tweets), ICR^b^for a subset of 125 tweets using kappa statistic (κ>.70), Pearson correlations between manual and automated coding, and chi-square to test changes over time, frequency tables, and text matrices with quotes illustrating the categories.
Scanfeld et al (2010) [27]	“antibiotic” and “antibiotics”	Random sample of 52,153 tweets. Tweets were posted weekly between March 13 and July 31, 2009 (n=1000 tweets were collected for analysis).	Cross-sectional survey approach using Q-methodology and CA (frequencies).	Pilot coding of 100 tweets, ICR for a random sample of 10% of the analyzed tweets using kappa statistic (κ=.73), frequency tables, and text matrices with quotes illustrating the categories.
Heaivilin et al (2011) [28]	“toothache”, “tooth ache”, “dental pain”, and “tooth pain”	Random sample of 4859 tweets over 7 nonconsecutive days (n=1000 tweets were collected for analysis).	Cross-sectional survey approach and CA (frequencies and descriptive statistics).	Pilot coding of 300 tweets, ICR using kappa statistic (κ=.96), frequency tables, and continuous text with quotes illustrating the categories.
Signorini et al (2011) [31]	“flu”, “swine”, “influenza”, “vaccine”, “tamiflu”, “oseltamivir”, “zanamivir”, “relenza”, “amantadine”, “rimantadine”, “pneumonia”, “h1n1”, “symptom”, “syndrome”, and “illness” and additional keywords (eg, travel, trip, flight, fly, cruise, and ship)	Two large data sets for tracking flu rate over time and location. The first data set consists of 951,697 tweets selected from the 334,840,972 tweets. Tweets were posted between April 29 and June 1, 2009. The second data set consists of 4,199,166 tweets selected from roughly 8 million tweets. Tweets were posted between October 1, 2009 and December 2009.	Quantitative CA (descriptive and advanced statistics).	Regression analysis and frequency graphs with respect to time.
McNeil et al (2012) [39]	“seizure”, “seizures”, “seize”, “seizing”, and “seizuring”	Random sample of 10,662 tweets from a period of 7 consecutive days. Tweets were posted between April 15 and April 21, 2011 (n=1504 tweets were collected for analysis).	Prospective qualitative CA.	Pilot coding of a 48-hour preliminary data set and interrater agreement (85.4%), frequency tables, and text matrices with quotes illustrating the categories.
Sullivan et al (2012) [40]	“concussion”, “concussions”, “concuss”, “concussed”, “#concussion”, “#concussions”, “#concuss,” and “#concussed”	Random sample of 3488 tweets over 7 consecutive days. Tweets were posted between 12:00 GMT^c^on July 23 and 12:00 GMT on July 30, 2010 (n=1000 tweets were collected for analysis).	Prospective observational study using qualitative CA.	Pilot coding of 100 tweets from a sample collected over a 24-hour period and interrater agreement, frequency tables, and text matrices with quotes illustrating the categories.
Donelle & Booth (2012) [41]	“#health” and “health” as a single word, part of a word (eg, health care)	Purposeful cross-sectional sample of 36,042 tweets. Tweets were collected over 4 consecutive days, from June 16, 2009 at 19:32 GMT until June 20, 2009 at 12:02 GMT (n=2400 tweets were collected for analysis; the first 100 tweets from the end of each hour of June 19, 2009, starting at 05:00 GMT for a 24-hour period).	Qualitative (directed and deductive) CA [20] guided by the Public Health Agency of Canada’s Determinants of Health framework.	Trustworthiness and validation of findings (interrater agreement, systematic data analysis, analyst triangulation, and verbatim data collection, and basic descriptive statistics). Data were presented through frequency graphs, text matrices, and continuous text with quotes illustrating the categories.
Robillard et al (2013) [29]	“dementia” and “Alzheimer”	Random sample of 9200 tweets for a period of 24 hours (starting February 15, 2012 at 3:35 pm) (n=920 tweets were collected for analysis in addition to a subsample containing 100 tweets generated by the top users).	Cross-sectional survey using CA [25,27] Statistical analysis (descriptive statistics) was used to characterize the composition of the sample.	Pilot coding of an initial set of 100 random tweets and frequency graphs and tables.
Lyles et al (2013) [42]	“pap smear” and “mammogram”	Cross-sectional sample of top tweets during a 5-week period. Tweets were posted between April and early May 2012 (n=474 tweets were collected for analysis).	Exploratory qualitative CA.	Pilot coding of 20% of collected tweets, ICR of 40% of collected tweets, interrater agreement, frequency graphs, text matrices, and continuous text with quotes illustrating the categories.
Bosley et al (2013) [32]	“cardiac arrest”, “CPR”, “AED”, “resuscitation”, “heart arrest”, “sudden death”, and “defib”	All identified resuscitation-related tweets from the keyword search. Tweets were posted between April 19 and May 26, 2011 (n=15,475 tweets were collected for analysis).	Quantitative CA (descriptive statistics).	Pilot coding of 1% of identified tweets, ICR using kappa statistic (κ=.78), frequency graphs and text matrices with quotes illustrating the categories.
Hanson et al (2013) [33]	“prescription drugs”	Random set of tweets posted by 25 identified social networks or circles. Tweets were posted between November 29, 2011 and November 14, 2012 (up to 3200 tweets per user were collected for analysis).	Quantitative CA of identified social circles	Pearson correlation coefficient of user interactions. Frequency tables and social network graphs.
Henzell et al (2013) [30]	“braces”, “orthodontist”, and “orthodontics”	Convenience sample of consecutive tweets posted over a 5-day period. Tweets were posted between September 3 and 7, 2012 (n=131 tweets were collected for analysis).	Qualitative (discourse) CA.	Continuous text with quotes illustrating the categories.
Myslín et al (2013) [26]	“cig*”, “nicotine”, “smoke*”, “tobacco”, “hookah”, “shisha”, “waterpipe”, “e-juice”, “e-liquid”, “vape”, and “vaping”	Random sample of tweets at 15-day intervals. Tweets were posted between December 5, 2011 and July 17, 2012 (n=7362 tweets were collected for analysis).	Infoveillance methodology [25], which includes iterative (manual) content and sentiment analysis.	Pearson correlations between manual and automated coding, chi-square to test changes over time, frequency graphs, and text representation diagrams.
Rui et al (2013) [34]	Not stated	Random sample of tweets posted by 58 health organizations (chosen randomly) within 2 months. Tweets were posted between September and November 2011 (n=1500 tweets were collected for analysis).	Quantitative (deductive) CA guided by the classic categorization of social support.	Descriptive statistics, ICR of 200 random tweets using Krippendorff alpha (.74), frequency tables, and continuous text with quotes illustrating the categories.
Zhang et al (2013) [35]	113 physical activity keywords generated from lists of published physical activity measures	A random sample of 30,000 tweets selected from a pool of one million tweets. Tweets were posted between January 1 and March 31, 2011 (n=4672 tweets were collected for analysis in addition to 1500 collected from this sample for further coding).	Quantitative CA (descriptive and advanced statistics).	Pilot coding of 100 tweets (separate from the final 1500 tweets) to calculate ICR (ranges from 0.83 to 0.98) using Holsti’s [9] method and frequency graphs and tables.
Park et al (2013) [36]	“health literacy”	Random sample of 1044 tweets. Tweets were posted during the time following time periods to construct a composite month: October 25–31, 2009; November 7–14, 2009; December 15–23, 2009; and January 4–10, 2010 (n=571 tweets were collected for analysis).	Quantitative CA based on Web reports on key Twitter features and previous literature in health communication and media studies.	Pilot coding, ICR of a subsample of 111 tweets using Holsti [9] reliability coefficient (.91), Krippendorff alpha (.85), and statistical analysis (frequencies and chi-square analyses and tables).
Love et al (2013) [37]	“vaccine”, “vaccination”, and “immunization”	Random sample of 6827 English-language tweets. Tweets were posted between January 8 and 14, 2012 (n=2580 tweets were collected for analysis).	Quantitative CA.	Statistical analysis (frequencies and chi-square analyses and tables).
Jashinsky et al (2013) [38]	Keywords and phrases created from suicide risk factors (12 identified factors)	All tweets (1,659,274 tweets) posted by 1,208,809 unique users over a 3-month period. Tweets were posted between May 15, 2012 and August 13, 2012 (n=37,717 tweets from 28,088 unique users were collected for analysis).	Quantitative CA (descriptive and advanced statistics).	ICR using kappa statistic (κ=.48), Spearman rank correlation coefficient, vital statistics, and text matrices with quotes illustrating the categories.

^a^CA: content analysis.

^b^ICR: intercoder reliability.

^c^GMT: Greenwich mean time.

**Table 2:** Twitter archive software used in the studies analyzing health-related Twitter posts (2010–2014).

Author(s)	Archive software used
Chew & Eysenbach (2010) [25]	Infoveillance system and Twitter API^a^
Scanfeld et al (2010) [27]	Twitter search engine
Heaivilin et al (2011) [47]	Twitter search engine
Signorini et al (2011) [31]	JavaScript application and Twitter’s API
McNeil et al (2012) [39]	Twitter search engine
Sullivan et al (2012) [40]	Twitter search engine
Donelle & Booth (2012) [41]	The Archivist (MIX Online, 2011) data collection software program
Robillard et al (2013) [29]	Twitter’s API
Lyles et al (2013) [42]	Twitter search engine
Bosley et al (2013) [32]	Twitter search engine
Hanson et al (2013) [33]	Twitter’s API
Henzell et al (2013) [30]	Twitter search engine
Myslín et al (2013) [26]	Twitter’s API
Rui et al (2013) [34]	ActivePython v2.7.2
Zhang et al (2013) [35]	Twitter’s API
Park et al (2013) [36]	Twitter’s API
Love et al (2013) [37]	Twitter’s API
Jashinsky et al (2013) [38]	Twitter’s API

^a^API: application programming interface.

The qualitative approaches to sampling techniques, such as purposeful and convenience sampling, were used in only 2 studies ([30,41]). The focus of most of these studies situated tweets as a primary source of information. The context of the tweets (eg, events or other Web-based information attached to tweets, if any, such as pictures, videos, user biographies, characteristics of active users, and social network communities related to that topic) was rarely mentioned. In 1 study [41], major world events were reviewed and summarized over the month of data collection, and an explanation of how those events related to the analyzed tweets was provided. However, the authors recommended the collection of a larger data set in order to examine the proposed inferences from world events in more detail. In another study [29], the characteristics of top users were described as frequencies in statistical graphs. Furthermore, when studies used CA with a cross-sectional survey design [27-29], they included mixed components of analyzed data, integrating quantitative data (quantitative strings or classifiers) with categories (codes or themes) developed inductively from the tweets. Units of analysis were inadequately described, and a single tweet was mentioned as a coding unit in only a few studies. For all studies, only publicly available data were used with no attempt to contact users.

Among the reviewed studies, all used a form of CA that was neither purely quantitative nor purely qualitative. Despite the fact that these two types of data were combined, no formal approach to mixing methods was described within any of the methods sections. With either approach chosen by the researchers there were mixed modes of analysis. Data were either imported and coded automatically (computer assisted) or imported automatically and coded manually (with human-assisted analysis). While the manual mode of CA can be used to qualify small amounts of coded data, the automatic mode may be used for large samples of either categorical or more quantifiable words or texts. The validation of results in these studies was based mostly on the pilot coding (also called trial coding [6]) or intercoder reliability (ICR) using Cohen kappa coefficient (kappa statistic) or Krippendorff alpha, which is more frequently used in both quantitative and qualitative studies. Descriptive statistics (mean, standard deviation, or correlation) or advanced statistics (regression analysis or chi-square) were used to validate the studies if the study’s primary approach was quantitative.

We propose that a blended research methodology that considers quantitative and qualitative perspectives in the study design and coding procedure would be fruitful for the advancement of CA methodologies. Further, an approach that allows for a combination of manual and computer-assisted coding through the most suitable supported software for the methodological approach of the study would be beneficial. A robust approach of this kind was not explained explicitly in these studies; we describe our proposed model for such studies is in the Discussion section.

## Discussion

### Model Development

Building on our review of the literature for key concepts, components, and data collection and analysis procedures of CA, and our appraisal of 18 health care social media studies, we propose the CCA model as a solid model for combining methods (quantitative and qualitative), coding procedures (inductive and deductive), and analytic modes (manual and automated) of CA. Our model is designed to address the mixed (quantitative and qualitative) nature of Twitter feed data in single or multiple-phase studies depending on the research aim of the phenomena under investigation. The model enables researchers to integrate methods and blend data in a single study—or a series of studies—using Twitter as a primary data source for analysis; it is a mixed-methods approach to CA research in the age of digital data. The CCA model integrates the major designs of mixed-methods research—the convergent, sequential, embedded, and transformative designs [48]—with the main features of each phase of CA. Our model can be divided into 3 phases: (1) preparation phase: research aim and keyword search and direction of the CCA model, (2) organization phase: sampling and data collection and coding procedure, and (3) interpretation and presentation phase: validation of study results and quality criteria and the use of computer software in CA.

**Figure 2:**
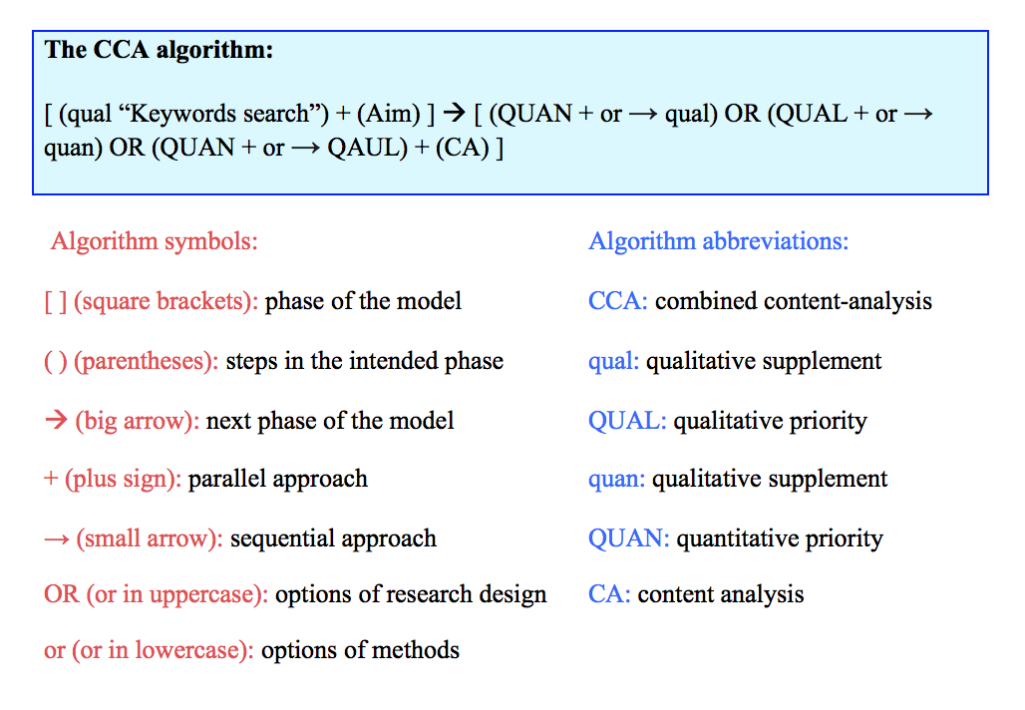
The combined content-analysis (CCA) algorithm.

Because text is always qualitative to begin with and the quantification of text alone is insufficient for successful understanding of content [7], quantitative and qualitative methods offer a more flexible alternative and dialectic integration of inductive (working from the data level) and deductive (working form the theoretical or hypothetical level) approaches. Given the nature of Twitter feeds, such an approach is more suitable than using CA without a clearly laid out and adapted methodology. The CCA model considers quantitative and qualitative perspectives either simultaneously (through a convergent parallel design) or sequentially, with either perspective serving as the predominant approach (through an explanatory or exploratory sequential design). Both quantitative and qualitative methods are embedded or nested within the predominant approach (through an embedded design). Those who are interested in ideology, political approaches, or theoretical frameworks (eg, critical theory, advocacy, or participatory research) aimed explicitly at societal change can use a transformative design with CA. The CCA design is useful when the researcher has more than 1 question best addressed through the use of multiple methods, or when the aim is to gain the best from each method by combining them to address a particular question. We propose an algorithm for the CCA model (see Figure 2).

**Figure 3:**
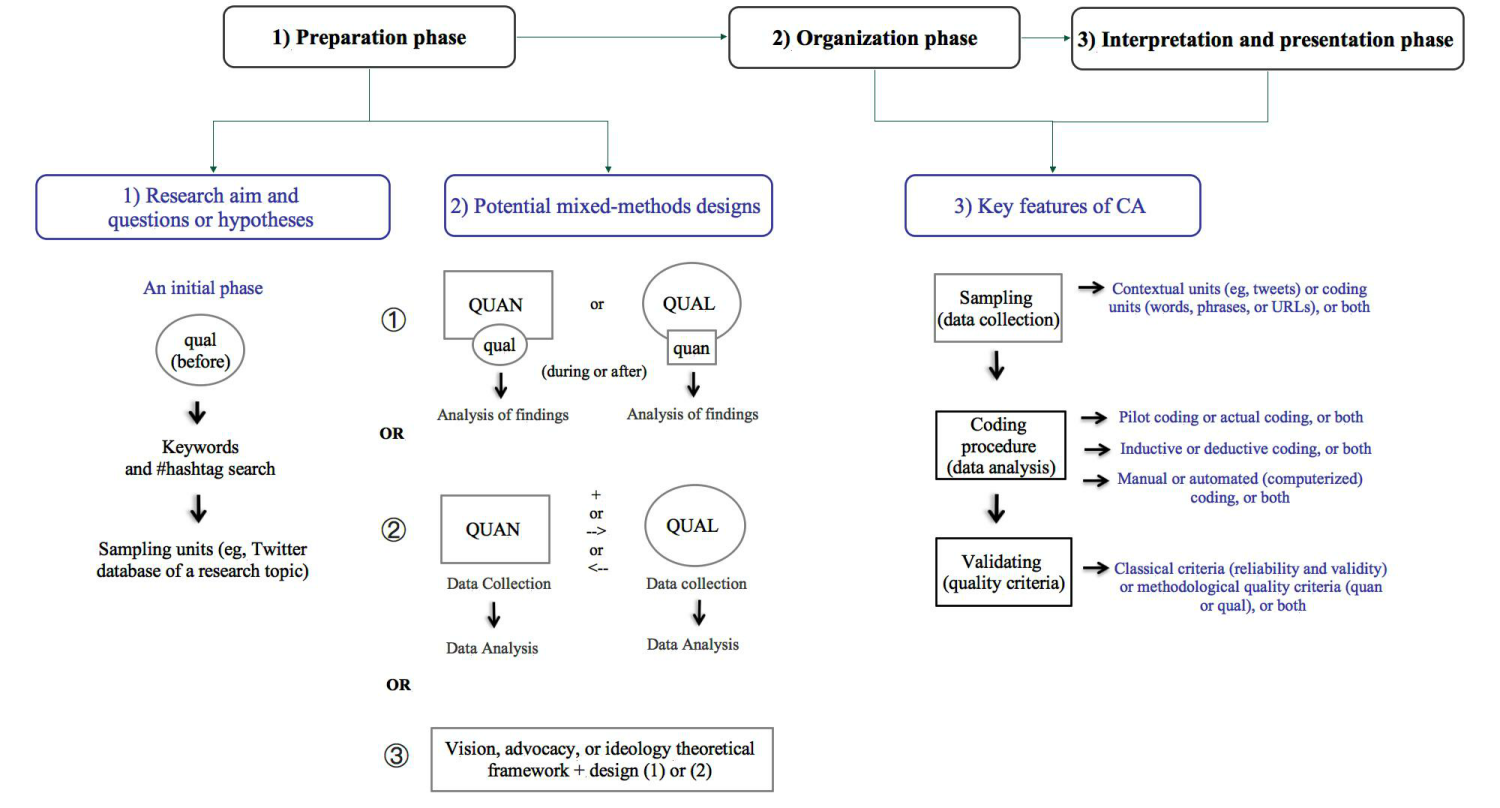
The combined content-analysis (CCA) model. CA: content analysis; qual: qualitative supplement; QUAL: qualitative priority; quan: quantitative supplement; QUAN: quantitative priority.

When referring to potential mixed-methods design, in the CCA algorithm we used the most common notations (abbreviations) used in mixed-methods literature [48]. For example, 4 letters indicate the quantitative “quan/QUAN” and qualitative “qual/QUAL” methods of the model. The relative priority of the two methods within a particular study or research project is indicated through the use of uppercase and lowercase letters. In addition, within the mixed-methods design, the plus sign indicates methods that occur at the same time, and a small arrow indicates methods that occur in sequence. “OR” in uppercase letters refers to potential options of mixed-methods designs, and “or” in lowercase letters refers to options regarding priority of methods (see Figure 3 for the CCA model). More details about the model are discussed in the next sections.

#### Phase 1: Preparation

Researchers interested in health care social media-driven data can use Twitter as a rich and useful data source to generate information related to their health topic. This way of collecting data may go beyond traditional data collection methods (eg, observations, interviews, or focus groups), and researchers may have a large amount of textual data that is shared by a diverse group of people in a social and natural platform. Analyzing Twitter-driven content such as tweets can be a productive way not only to analyze text, but also to evaluate discourses surrounding health and disease-related issues [25,27]. Unless a Twitter account is protected by its user, Twitter content is largely public and freely accessible through the Twitter website or mobile and tablet apps (see Multimedia Appendix 1A for a Twitter overview).

Twitter features a search function (eg, keyword or hashtag search) to filter status updates that meet particular search criteria. Archive software is also available to search, track, store, and retrieve targeted health topics from collected tweets by date, time, and possible geographic location. Because reading any form of text, even using a technical search, is fundamentally an interpretive process regardless of its numerical outcomes [7], there is a need for a flexible model that takes into account qualitative as well as quantitative data to respond to multiple research aims. In the CCA, identifying the research aim, including the qualitative keyword search, and identifying the research direction are the 2 initial steps of the preparation phase of the model.

##### Research Aim and Keyword Search

Before conducting a study on health tweets, several factors are important for researchers to consider in deciding what CA approach to use. First, it is essential to confirm that data on their topic have been tweeted (preliminary search for data) and to determine the time frames or periods of time when this has occurred. Some Twitter databases may be created in response to a specific event (eg, an Alzheimer awareness day or month); the data cannot be interpreted well if that event (the context of the data) is not taken into account in the analysis. Discussions on specific health topics may not be established yet, and the number of tweets may be insufficient to facilitate analysis. Searching for health-related keywords in Twitter is the first step for any Twitter-driven study using CA. This step is common to traditional summative CA studies and mirrors the first part of the CCA model equation ([ (qual “Keywords search”) + (Aim) ] →), which is usually qualitative in nature because it is done manually. However, the Twitter database itself may be collected directly from Twitter (eg, Twitter’s advanced search), downloaded from chat recaps (eg, Twitter chat transcripts) using particular health care social media websites (eg, the Healthcare Hashtag Project [49]; see Multimedia Appendix 1B for examples of elder care tweet chats), or captured through an automatic mode using a Twitter archive software package (eg, Analytics for Twitter in Excel, Microsoft; or NCaptur in NVivo, QSR International). More advanced automated approaches that use algorithms and dictionaries or machine-learning approaches can be used to filter tweets. We briefly describe the use of software in assessing CA below. This step of the CCA model should also include establishing a clear plan and study objectives (eg, hypotheses or questions) that would meet the needs of the research, which is an important factor in the next step: choosing the appropriate methodology for conducting the research.

##### Direction of the CCA Model

Availability of data and worded objectives will help researchers choose the study, data collection, and analysis approaches to use. To make a final decision on study approach, it is important for researchers to consider which CA approach will be helpful in achieving their desired results. For example, researchers might ask the following questions. Should we test hypotheses by counting words (a single word), the co-occurrence of words (word-to-word), or text as a whole in the targeted tweets? Should we explain counted results using descriptive or inferential statistics and then integrate additional qualitative information (eg, QUAN + or → qual)? Should we try to understand the environment surrounding tweets (text and related context) by asking questions and seeking answers within the data and then support the answers using descriptive statistics (eg, QUAL + or → quan)? Are both numbers or hypotheses and words or questions equally important in understanding the big picture (eg, QUAN + or → QAUL)? Are we interested in an interpretive analysis of the content and, if so, what qualitative methods can best inform the design and analysis? By considering all of these factors researchers can choose an appropriate direction (and potential assisted software) for CA as per the second part of the CCA algorithm [ (QUAN + or → qual) OR (QUAL + or → quan) OR (QUAN + or → QAUL) + (CA) ].

#### Phase 2: Organization

The last part of the equation, “+ (CA)”, includes the key feature of successful CA, which moves from selecting the sample of content, establishing the coding process, and developing or testing category schemes to determining the quality criteria of study results. We provide these steps and explanations of how combined mixed-methods approaches to CA (as shown in the CCA algorithm) can be applied to the analysis of Twitter feed content in this section.

##### Sampling and Data Selection

Although in all potential approaches—that is, “(QUAN + or → qual) + (CA)”, “(QUAL + or → quan) + (CA)”, and “(QUAN + or → QAUL) + (CA)”, —researchers sample the text or “universe” [2,7] from a Twitter database or transcript of written tweets with or without attached material, such as pictures, URLs, or videos, there is no previous research on a validated sampling method for Twitter data [25]. This methodological gap poses a challenge for researchers in selecting the appropriate sample of tweets and defining its related context. Furthermore, there is a need for translation of Twitter texts into CA sampling terminology. On the basis of Neuendorf’s [43] typology of CA texts, CA researchers should take into consideration the number of participants or setting of the messages. Twitter posts can be individual messages (for 1 user or between 2 or more users), interpersonal (group) messages, or organizational messages [50]. All three kinds of messages can help to define the appropriate context of collected tweets. In addition, Twitter posts consist of three types of CA units: sampling units (units of selection), contextual units (the largest textual units of analysis in a category), and coding or recording units (the smallest units of analysis or units of description). All three units need to be conducted within a suitable multistage sampling frame that differentiates CA from other methods of data collection. For example, the extracted Twitter database or selected transcripts of Twitter chats on a specific topic within a limited time frame can be identified as sampling units that identify the population and establish the basis for inclusion and exclusion criteria. The content of a single tweet can serve as a contextual unit. The recording or coding units can include different levels of units in a single tweet, such as words, phrases, URLs, pictures, or videos, that are the basis for establishing the coding schemes. A suitable selection and precise description of the different kinds of CA units can help with the evaluation of reported results in later steps of CA [4,8].

Despite the gap in the social media methodology literature about sampling, the CA literature follows the general direction of research paradigms [5,7]. Cohen et al [8] argue that the rules of sampling human subjects can be applied to sampling documents. Building on their argument, the same rules can be applied to social media data taking into account the nature of massive Web-based content and the sampling frame of CA. Based on existing paradigms, the number of tweets (many or few) in a Twitter database that are purposefully tracked or retrieved (using specific keywords or hashtags) and chosen for analysis, the method of selecting the tweets (probability or nonprobability sampling) within the database, and the time period of tweets (cross-sectional or over time) all affect the subsequent steps of CA. As a result, the “(QUAN + or → qual) + (CA)” approach would be appropriate for evaluating a random (representative) sample (thousands or millions) of tweets that are randomly selected, where each tweet has an equal chance of selection. In this case, there is a chance for generalizability, reproducibility, and making valid inferences from the text (the manifest content of analyzed tweets) to the universe (a broader representation of tweets) based on valid statistical conclusions with less focus on the context of the tweets. With random samples, it is also essential that researchers know all units (the universe) in the population (all Twitter database or potential sample of tweets related to the topic). Other probability sampling techniques, such as stratified sampling, can also be applied, when a range of dates or points in time may be set to focus on a random subsample of tweets. Contextual units and analysis units can be the same (tweets) in some cases (eg, when there is no material attached to the tweets and the whole tweet is used to establish categories); however, a unit of analysis cannot be larger than the unit of context. It is important to carefully define all three kinds of units, because different levels of units may influence the credibility of CA [4] and require different levels of reliability [7,8]. In addition, the sample can be completely randomized before initiating the coding process; however, the supplemental or sequential “qual” part in this approach can work, and, if needed, collecting a small (purposeful) subsample (eg, tweets of top or active users or chat managers) can assist in refining the codes developed from the random sample during or after the dominant “QUAN” analysis.

On the other hand, with the “(QUAL + or → quan) + (CA)” approach, the focus is on the transferability rather than the generalizability of results. As such, researchers can purposefully collect a sample of tweets (hundreds) within the tweets database that is unique to specific users (eg, regular users or chat managers of a specific topic identified by an elder care-related hashtag), events (eg, an elder care-related event), or researchers’ assumptions about such tweets. Nonprobability samples, such as purposeful, convenience, and other types of qualitative samples, allow for the collection of important interpretive data and for the consideration of research questions that acknowledge the contexts, meanings, emphasis, and thematic dimensions of the topic. For example, a researcher might select his or her purposeful sample based on selected tweets of a popular health care community on Twitter (eg, #AlzChatUS). The selection of data may continue throughout the coding phase. Once the researcher establishes a rationale for specific tweets (which are likely to involve purposive, convenience, or other nonrandom sampling methods), the dominant direction of the study will no longer be quantitative, unless the rationale is combined with a random sampling method for the inclusion of tweets in the study. For instance, if researchers choose to analyze the random tweets of top users on an Alzheimer awareness month or day, the “(QUAN + or → QAUL) + (CA)” approach might lead the study, because the tweets, their environment, and specific (top) users are important. Regular tweets about Alzheimer disease from users tweeting on this subject may differ from tweets and users on Alzheimer awareness month or day. If researchers want to choose their sample purposefully (tweets of Alzheimer awareness month or day) but also want to track the changes of tweets over time (eg, in 2010, 2012, and 2013), this also means that the two approaches lead the study equally, because the aim is to track changes over time related to a specific event or Twitter context. It is important, however, to note that there is a potential for rich data within the structure of the social network from which the textual information is derived—information that may best be understood through an application of social network analysis. Such analyses are, however, beyond the scope of this paper. Further information may be found in Gruzd and Haythornthwaite [51].

##### Coding Procedure and Data Analysis

Establishing coding categories is one of the most fundamental steps in CA, especially for checking the quality criteria of the study, such as trustworthiness [4]. As explained in Figure 1, while coding in the “(QUAL + or → quan) + (CA)” approach can be inductive or deductive, “(QUAN + or → qual) + (CA)” is always deductive, and researchers may rely on coding schemes devised by other researchers or theories. Categories provide the structure for grouping the recording or coding units and can be considered the heart of CA, because when there is a large amount of textual data, text can be reduced into fewer and more abstracted categories or concepts [8] either to develop a theory or to support an existing theory. Therefore, categories must relate to the research goal and be designed to truly respond to the research questions [2,46]. As Berelson [11] pointed out, successful CA is seen in studies with well-structured categories that are strongly related to the research goal of the analyzed content. To systematize and organize CA, structured and well-defined categories are important. Categories that are exhaustive, mutually exclusive [9], and independent allow all relevant items (eg, scientific information, news, and personal statements) in a tweet to be placed into a single category. With deductive approaches to coding, the coding scheme is developed before the coding begins in order to test hypotheses or retest models or theories (eg, [41]). If adjustments are made during coding, items already coded must be recoded with the revised scheme. In contrast, in inductive coding the coding scheme is usually guided by the study questions and developed in the process of close and iterative reading (and sometimes sampling new tweets) to identify significant concepts and patterns (eg, [42]). Furthermore, researchers can record memos of their comments during the analysis, and these memos can be used in the inductive coding process. The overall process of inductive coding may suggest new questions that were unanticipated at the start of the analysis and that can be added to the study questions. In addition, inductive coding can be guided by more specific qualitative approaches, such as discourse analysis, rhetorical analysis, or ethnography [7]. For example, using qualitative coding with discourse analysis can take the analysis of tweets to a more advanced level than just coding the words to include symbols and related emotions (eg, [30]). These qualitative approaches can be modified to fit the purpose of collecting digital data. The integration of both the inductive and deductive procedures is sometimes called abduction [44], in which theory-oriented and data-oriented categories are generated simultaneously or sequentially.

#### Phase 3: Interpretation and Presentation

It is suggested that CA has the potential to be a valid and reliable tool to summarize extensive content if it is conducted carefully with clear and understandable results and well-described categories. This strength of the research is enhanced when researchers explain how they matched the reported results in their study with the study’s aim, questions, and hypothesis. This matching can be done with the use of quality criteria of CA. When considering the evaluation of CA results, there are two ways to ensure the rigor of a CA study: (1) using classic criteria to determine valid and reliable CA, and (2) using specific criteria to assess quality within the dominant research paradigm used. With the first way, while validity and reliability concepts can be used with quantitative CA, QUAN-dominant study and results can be presented through basic and advanced statistics (eg, percentages, probability, or inferences) that allow for objectivity and replication. Credibility, transferability, dependability, conformability, and other areas for ensuring trustworthiness [52] can be used in QUAL-dominant studies [5], and a group of these concepts can be used if the QUAN and QUAL approaches are used equally in the study. In the next sections, we discuss the issues associated with the validation of CA results. Furthermore, we discuss how the use of computer software can help with the preparation, analysis, interpretation, and validation of CA results.

##### Validation of Study Results and Quality Criteria

Schreier [6] claimed that in validating the results of CA, there are no clear divisions between approaches. Terms such as rigor, reliability, and validity are usually used with either the qualitative or quantitative approach to CA, despite their positivistic origin from quantitative research or constructivist origin from qualitative analysis. Therefore, reliability, in particular ICR, is often used as a classic quality criterion for both qualitative and quantitative CA. The use of ICR is consistent with the most common use of CA to evaluate study results in the literature. With ICR, at least two coders usually conduct the analysis, especially if the deductive procedure is used for either QUAN or QUAL as the predominant approach. ICR reliability is one type of reliability that is often used to insure the reproducibility of a coding matrix in deductive coding of data [7], that is, the likelihood that all coders under different circumstances will code the same group of items the same way (consistency and agreement between coders). This ensures that categories are sufficiently defined so that all coders reach the same conclusion.

Another way to test ICR reliability is to use reliability checks before conducting the analysis, which often entails pilot coding (trial coding) or pretesting categories several times before the actual coding. Pilot coding involves coding a small portion of the tweets to be analyzed or all tweets generated before selecting the sample (all retrieved sampling units). Such a pretest can enable researchers to determine whether the categories are clearly specified and meet the requirements, that the coding instructions are adequate, and that coders are familiar with the data and are suitable for the job. It is recommended that with a QUAN-dominant study, the sample of pilot coding should be different from the sample of actual coding. In contrast, if the QUAL-dominant approach is used, the sample of pilot coding should be a subset of the sample of actual coding [6]. Once high reliability standards of the pilot phase are met (all categories are pretested and critically examined and modified), the actual coding can begin. High ICR reliability can strengthen the validity of the coding procedure; however, reliability is not guaranteed [9]. With a large amount of text, the comparison of results between coders becomes more difficult. Therefore, both ways of testing ICR are needed to ensure reproducibility and reliability as a way of producing stability of results over time [7].

With an inductive coding procedure, on the other hand, reliability checks between coders may not be helpful when an in-depth (line-by-line) analysis and iterative process is required. According to Elo et al [4], qualitative coding takes time and requires going back and forth with the data several times to ensure credibility and confirmability of findings. Therefore, it is suggested that 1 researcher can code the data, and experts in the targeted topic, participants, or readers can evaluate the coded data. This quality criterion may be referred to as intracoder reliability or member checking. As a requirement for intracoder reliability, the coding scheme should have clear definitions, straightforward instructions, and unambiguous examples or quotes to help assess the quality of results [4].

Validity with CA may refer to the representation of the intended concept [43] and that the data of the study has good face validity [4]. Construct validity is also significant to CA, meaning that categories truly test the proposed hypotheses or answer the study questions. In addition, mutually exclusive categories should be maintained to ensure validity and proper statistical inferences in QUAN studies. In a “(QUAN + or → qual) + (CA)” study, sampling validity is strongly related to the selected sample [7]. A biased and unrepresentative sample would hurt the study. Although with a “(QUAL + or → quan) + (CA)” study all decisions regarding sampling must be justified and the sampling strategy must be explicitly described (systematic sampling procedure), in qualitative CA research the important criterion is not numeric, but conceptual consistency between observation and conclusion. Findings are confirmed by looking at the data, not the sample or coder(s), to determine validity. If the data support the conclusions, the study is valid. Thus, validity checks are more important than reliability checks in this case [6].

Representing the results linked to the quality criteria of CA, particularly showing the connection between the aim of the study and the reported data [4], is important. Difficulties in structuring the data are related to unsuccessful CA analyses or to challenges that researchers face in the abstraction process. In contrast, clear and systematic representation of the data corresponds to successful analysis [15]. Conceptualization of coded results may differ according to the CA design used. For example, researchers may use numbers or percentages, either in simple tabulations or in cross-tabulations, to show relationships, but they may also rely on the gradual accretion of details within the textual presentation without resorting to numbers. While represented quotations, figures, and flowcharts of coded concepts are recommended in the QUAL approach to CA [4], frequency tables, percentages, and more advanced statistical values are recommended for the QUAN approach to CA [7].

##### Using Computer Software in CA

This section summarizes how technology can be used to facilitate different approaches of CA. As mentioned, the main idea behind CA is to break down a large amount of text into small codes, nodes, categories, themes, or concepts by making links between those concepts to support an emergent theory or test an existing theory [8]. The use of software for CA depends on many factors that can only be decided based on each individual project [1]. The number of researchers and their level of experience with the chosen methodology, the amount of coded text, the study’s financial plan, and the availability of and preferences for computers are important factors in determining the mode of CA. In the CA literature, software packages have been used to assist the process of coding [7,44], saving time and handling the hard work associated with manual coding of textual data (eg, highlighting sentences, writing analytical memos, and retrieving and connecting codes). Another reason is that computerized CA may enhance the validity and reliability of the coded data by filtering tweets, classifying codes, managing the sampling of text, and producing the same results across human coders each time they run the data [1,7,44].

In aiding CA, the software can be classified into two types: (1) computational software packages, such as text mining and statistical software packages [43], and (2) qualitative analysis software, such as computer-assisted/aided qualitative data analysis software (CAQDAS) packages [44]. Under each classification there are various types of packages and different analysis techniques. The role of software used may vary according to the aim and methodological plan of the study. For example, the role of software in a qualitative CA study is not to perform the analysis; rather, it is limited to the facilitation of data management and the analytical process carried out by the researcher. In contrast, the software for quantitative CA can do a lot more than aid in the analysis, as it can automatically code the words that have been decided in the dictionary of key terms created by the researcher [6]. Table 3 provides a nonexhaustive list of available software packages and their reference websites. It is recommended that researchers compare and contrast software features, examining the utility of software based on the study methodology and type of data gathered for analysis. In addition, training sessions for computerized coding is required for coders to deal with the complexity of data analysis, to reduce coding errors, and to ensure that the produced results answer the research questions [8].

**Table 3:** Selected software to aid content analysis.

Software (source)		Web address
**Computational software packages**		
	Analytics for Twitter for Excel (Microsoft)	www.microsoft.com/en-us/download/details.aspx?id=26213
	twitteR (The Comprehensive R Archive Network)	cran.r-project.org/package=twitteR
	Tweet Archivist (Tweet Archivist)	www.tweetarchivist.com
	Twitter Analytics (Twitter)	analytics.twitter.com/about
**Qualitative and integrative software packages**		
	CAQDAS^a,b^Networking Project (University of Surrey)	www.surrey.ac.uk/sociology/research/researchcentres/caqdas/support/choosing/
**Other**		
	Text Analysis Info (Social Science Consulting)	textanalysis.info/pages/text-analysis-software---classified.php

^a^CAQDAS: CAQDAS (computer assisted qualitative data analysis) networking project.

^b^For example, ATLAS.ti, NVivo, MAXQDA, Dedoose, HyperRESEARCH.

In addition to the benefits of computerized coding listed above, software can be used to capture multiple types of data, such as multimedia data (eg, sounds and videos). On Twitter, for example, tweets can be coded manually or by data-analysis software depending on the leading approach chosen, length and format of the text (tweets), and the researchers’ aims. It is suggested that with limited qualitative data, manual coding provides a better understanding of the meanings between the lines [15]. CAQDAS software programs (eg, ATLAS.ti, NVivo, and MAXQDA) can be used for larger texts to make CA more manageable and ordered. On the other hand, Twitter’s application programming interface streaming [53] and quantitative statistical software (eg, R or Analytics for Twitter Excel add-ins) can be used with more advanced statistical analysis of tweets, such as multivariate analysis and factor analysis. With the use of the research approach suggested through the CCA model, numerous software packages can be used to aid the collection and analysis of data, especially when applying algorithmic approaches to CA (eg, machine-learning approaches [54]), where, for instance, software (eg, Python packages [53]) can be used to specify models and identify patterns to extract the content computationally based on a certain classification and categorizing of highest probability (statistical classification). Other software (eg, CAQDAS packages) can be used to code the gathered contextual content with support of human coders (eg, [55]).

### Conclusions

CA is a prevalent methodology used to analyze health care social media-driven content, such as Twitter feeds. With the digital revolution of social networking platforms, Twitter has become a common source for online discussions on health issues; thus, health researchers need to become familiar with a structured model of CA that can respond to the nature of the retrieved digital data and the varied purposes of their studies. This paper reviews the general and health care literature of CA and evaluates how CA was used in Twitter-driven studies between 2010 and 2014. The CCA model is suggested as a new research framework that takes into account the various dimensions of the CA research methodology in a way that allows for mixing methods, procedures, and modes and components of CA. Thus, the CCA model will be useful in designing new studies (as a structured model) and evaluating existing studies (as an outline or checklist) that require or use various types or multiple modes of information within a single coherent model. The model integrates the main features of CA with the most common designs of mixed-methods research to facilitate the application and evaluation of studies that intend to use CA to analyze social media-driven content related to the researched phenomenon.

## Appendix

Multimedia Appendix 1 (A) Twitter overview. (B) Examples of eldercare tweet chats [51].

## Abbreviations

CA: content analysis
CAQDAS: computer-assisted/aided qualitative data analysis software
CCA: combined content analysis
ICR: intercoder reliability
QUAL: qualitative priority
qual: qualitative supplement
QUAN: quantitative priority
quan: quantitative supplement
