# Supplementary figures and images for: Toward a Mixed-Methods Research Approach to Content Analysis in The Digital Age: The Combined Content-Analysis Model and its Applications to Health Care Twitter Feeds
Source: J Med Internet Res. 2016 Mar 8;18(3):e60. doi: 10.2196/jmir.5391 (PMC4804105; doi:10.2196/jmir.5391)

(A) Twitter overview.

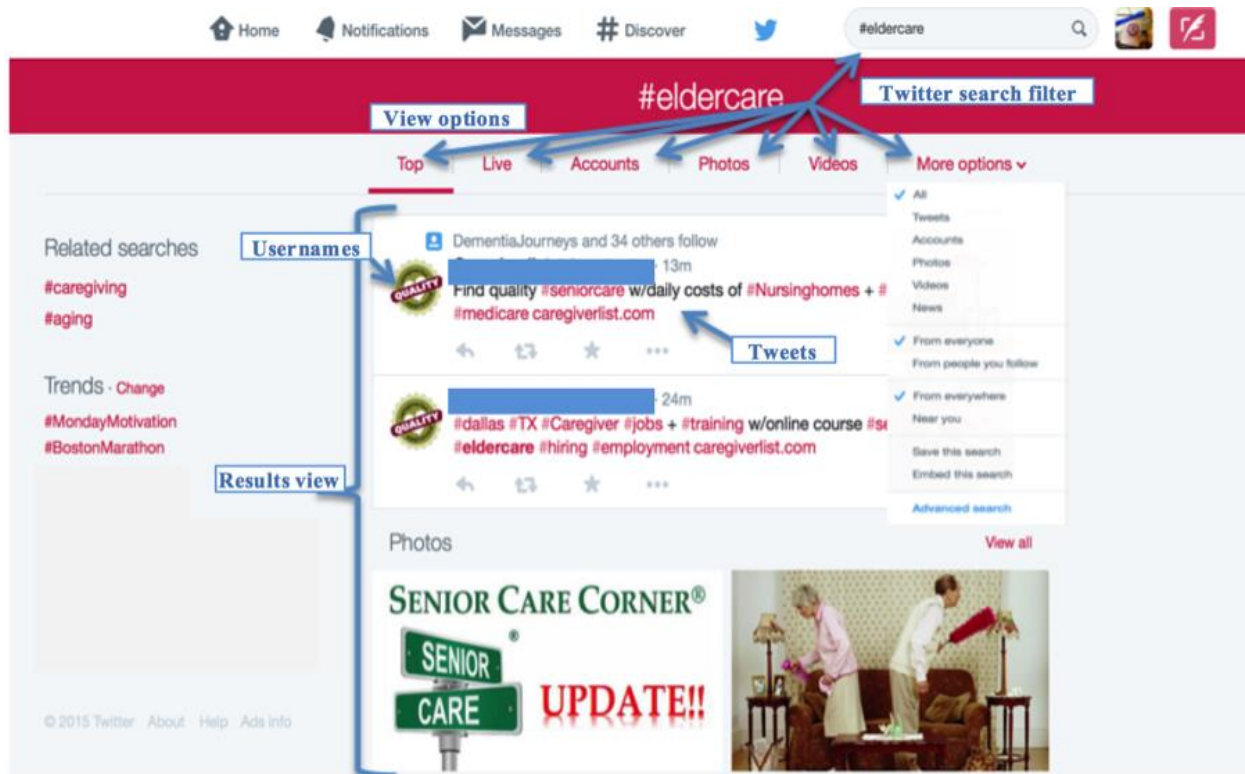

(B) Examples of eldercare tweet chats [51].

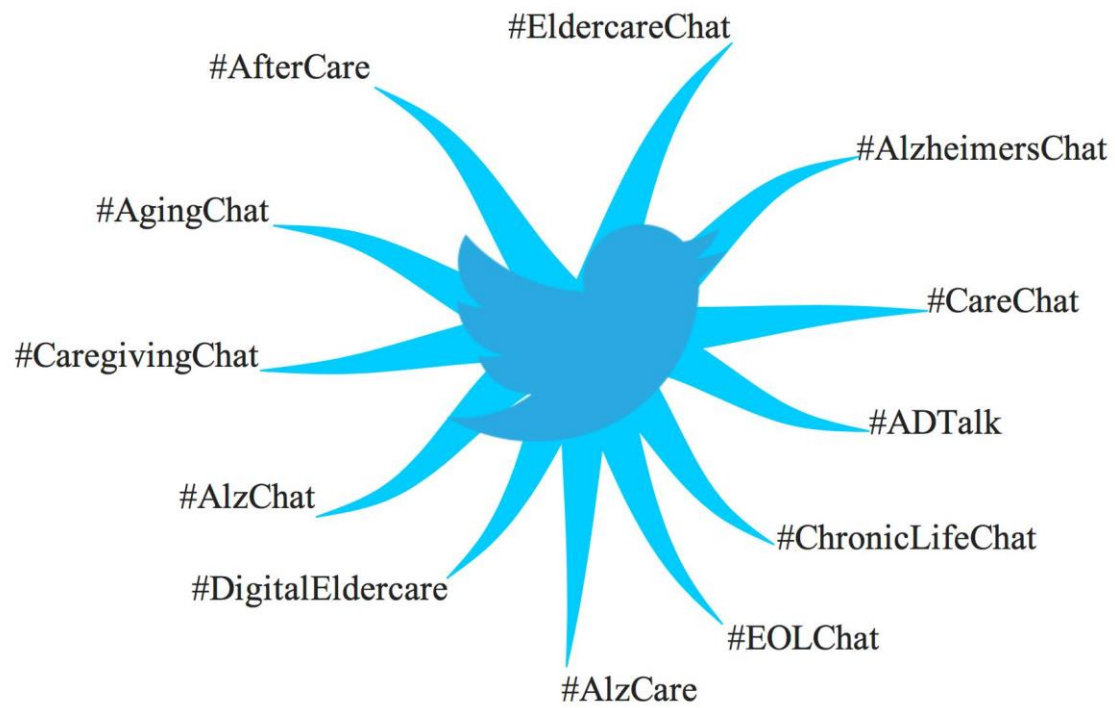

Supplement: Multimedia Appendix 1 [file jmir_v18i3e60_app1.pdf]
